# The Risks to Patients Associated with Using Artificial Intelligence Tools in Pharmacy Practice: A Scoping Review

**DOI:** 10.3390/pharmacy14050113

**Published:** 2026-08-03

**Authors:** Tracy Zhang, Minh-Hien Le, Noah Zlotnik, Amanda Yee, Anjali Patodia, Zubin Austin

**Affiliations:** Department of Pharmaceutical Sciences, Leslie Dan Faculty of Pharmacy, University of Toronto, Toronto, ON M5S 3M2, Canada; mh.le@utoronto.ca (M.-H.L.); noah.zlotnik@mail.utoronto.ca (N.Z.); amandabear.yee@mail.utoronto.ca (A.Y.); anjali.patodia@utoronto.ca (A.P.)

**Keywords:** artificial intelligence, pharmacy practice, patient safety

## Abstract

Artificial intelligence (AI) tools are being used in pharmacy practice in a variety of ways, such as enhancing the safety and efficiency of dispensing, compounding medications and helping pharmacists identify drug–drug interactions. The further development and use of these innovative technologies will be necessary to ensure patients can continue to access high-quality care amidst a growing health human resource crisis. However, in the field of pharmacy practice, little is known about actual or potential risks these tools pose to patients. A scoping review was conducted to map these risks. A database search of Ovid Medline, Ovid Embase, Ebsco CINAHL, and Web of Science, and a grey literature search were conducted. Three major potential risks were described in the literature: inaccuracies associated with AI outputs, privacy and data security concerns, and risks associated with algorithmic bias. Despite these potential risks, there is still a large gap in the literature regarding the study of risks of AI in pharmacy practice. While characterizing all risks associated with this rapidly changing technology may be impossible, further exploration of risk, perhaps using novel approaches to understanding risk itself, is warranted if these technologies are to be adopted responsibly and used safely.

## 1. Introduction

Artificial intelligence (AI), broadly defined as machines that can do tasks typically requiring human intelligence, is revolutionizing many industries, including pharmacy. Pharmacy professionals and pharmacy regulators continue to wrangle with how to safely and responsibly adopt AI tools into practice [1,2]. AI has provided a multitude of benefits when used in a patient care context. Large language models (LLMs) have helped to coach patients through how to quit smoking, AI scribes save clinicians time by writing documentation notes for them, and other AI systems are even saving patient lives via early warning systems designed to alert clinical staff to assist patients at risk of needing intensive care to prevent deterioration [3,4,5]. Sustaining our healthcare system is becoming increasingly difficult due to an ever-ageing and chronically ill population and a shortage of health human resources. Innovative technologies like AI are vital to ensuring individuals can continue to receive high-quality, accessible healthcare [6].

However, using AI does not come without challenges. LLMs have a propensity to ‘hallucinate’ or fabricate information that is completely untrue [2,7]. Given their focus on modelling language, they also perform poorly when asked questions related to numbers and math, with many models unable to discern how many ‘Rs’ are in the word ‘strawberry’, for example [7]. While this may seem relatively innocuous or even mildly entertaining, there are challenges to using AI that have posed real harms to individuals. Chatbots are programmed to be sycophantic, or highly agreeable with users. Although not a formal diagnosis, a phenomenon known as ‘AI psychosis’ has been documented in media reports, where users develop or even act on delusions and paranoid beliefs that are amplified or often encouraged by the chatbot [8,9]. The ways in which users interact with generative AI systems may expose existing individual vulnerabilities to psychosis [10]. Since the performance of many AI systems depend largely on the data used to train and validate the algorithms, there are also risks associated with algorithmic bias. This has been especially salient in law enforcement, where the use of facial recognition technology has led to wrongful convictions because the algorithm performs poorly in recognizing faces of people of colour [11].

The literature related to AI has expanded exponentially over the past few years [12,13]. In addition to published papers, new conference abstracts, media reports and commentaries, and guiding principles and statements from regulatory bodies have discussed its various beneficial uses as well as some associated risks and challenges [13,14]. In the field of pharmacy, the literature related to AI has seen a similar explosion. There are papers discussing its use in drug development, drug–drug interaction checkers, robots assisting with medication dispensing and delivery, personalized medicine and tools to enhance patient medication adherence [12]. However, much of the literature in pharmacy practice focuses on how AI can be used beneficially to improve operations and patient care. Despite the growing knowledge regarding risks of using AI in other contexts like law enforcement and psychiatry, very little is known about the risks, including both theoretical or potential risks these tools pose to patients as well as risks that have caused patients harm when used in a pharmacy context [12,13,14].

Pharmacists are amongst the most accessible healthcare providers, and have been increasingly called upon to provide high-quality, community-based care to individuals [15]. Across Canada, pharmacists have been given expansions in their scopes of practice, allowing them to prescribe medications to treat minor ailments and to provide important vaccinations to better meet the needs of Canadians struggling to access primary healthcare [16,17]. Pharmacies have also traditionally been hubs for innovative technologies to promote more efficient and safer care. Automated dispensing machines are used routinely to more accurately dispense pills and compounding robots aid in the mixing of high-risk drugs like chemotherapy [11]. When it comes to AI, many of these tools used in pharmacy practice are considered ‘human-in-the-loop’, where a pharmacist must validate the AI’s output before it can be operationalized [2,18]. While this theoretically helps to ensure that incorrect outputs and therefore harms to patients can be mitigated before they occur, there are already vast amounts of other electronic alerts and alarms presented to a pharmacist and ‘alert fatigue’ is a phenomenon that clinicians already face [2,19]. Alert fatigue increases the risk of automation bias whereby humans in the loop may act more passively, or ‘on the loop’, agreeing blindly with AI outputs and merely ‘pressing go’ without much thought, allowing potential mistakes to slip by [20,21]. There has also been a push towards developing increasingly agentic, autonomously acting AI systems, referred to as ‘human-out-of-the-loop’ systems, and when these appear in pharmacy practice, any safeguards associated with having a pharmacist in the loop will disappear [22].

There is an urgent need to better understand both the potential and actual risks associated with using AI in pharmacy practice. There is a relatively poor understanding of risks in this area compared to in other industries and patients stand to be potentially harmed by the outputs of these tools. Also, the use of AI by pharmacists is largely unregulated right now [2,23]. Different clinicians may choose to use these tools in different capacities and there is currently no guidance from pharmacy professional regulatory bodies on the safe and responsible use of these tools. In fact, there has been a general hesitancy amongst healthcare professional regulatory bodies to impose strict regulations around the use of AI for fear that it may stifle necessary innovation [2]. There is a ‘central tension’ in AI regulation between ensuring that there are sufficient regulatory guardrails to protect vulnerable patients from harm, whilst still allowing for the development and adoption of necessary and important innovative solutions [2,24]. Characterizing and studying risk is critical for regulators to develop appropriate safeguards in this current ‘wild west’ of largely unregulated and unstandardized use of AI [25,26]. In this timely paper, we have reviewed the literature to understand the risks to patients associated with using AI in pharmacy practice. We have identified three major risks to patients, as well as a general gap in the literature when it comes to studies focused on risk in pharmacy practice.

## 2. Materials and Methods

To gain a better understanding of the risks to patients associated with using AI in pharmacy practice, a scoping review of both the published and unpublished literature was conducted. A scoping review was chosen methodologically because it aims to map a relatively unknown area of the literature [27]. This methodology lends itself well to the rapidly evolving nature of the field of AI by considering both the published and unpublished literature. An Ovid Medline search of the published literature was conducted prior to embarking on this work and registering our protocol; to our knowledge, this is the first review of this kind focused on pharmacy practice. This scoping review was conducted in accordance with the JBI methodology for scoping reviews and follows reporting guidelines as outlined by PRISMA-ScR [28,29]. The protocol for this review was registered in Open Framework Science [30].

A search of four databases was conducted: Ovid Medline, Ovid Embase, Ebsco CINAHL, and Web of Science. All citations were imported into Covidence (Veritas Health Information, Melbourne, Australia), a systematic review software, and deduplicated. Two independent screeners, TZ and NZ, conducted title and abstract screening according to the eligibility criteria (Table 1). After title and abstract screening, the full text of all articles was reviewed independently by TZ and one of NZ or AY to judge its relevance to the research question and inclusion in this review. For title and abstract screening and full text screening, any disputes were discussed amongst the two parties until a consensus was reached.

Our search intended to capture studies describing the many forms of artificial intelligence but did not extend to other aspects of digital health technology or simple automation. We used an operational definition of AI as any machine or computer that is able to simulate human learning, comprehension, problem solving, or decision making [1]. We also aimed to be inclusive of all types of pharmacy practice, capturing a range of services or activities that a pharmacist may do when providing direct patient care in various pharmacy practice settings. The concept ‘Risk to Patients’ was not included in our search but rather used as a criterion for screening articles for inclusion. Our database search was conducted on 20 March 2025, and our full Medline search strategy can be found in Appendix A.

Given our focus on risks to patients, non-patient-facing aspects of pharmacy, such as drug discovery and research and development, were excluded. Researchers allowed for a broad definition of ‘Risk to Patients’ in order to capture any and all risks discussed in the literature. Risks were considered to be any harm or negative outcome, either theoretical or something that has actually happened, explicitly stated in papers as impacting patients.

A search of the grey literature was also conducted by TZ and AP to ensure comprehensiveness and that relevant information which may not be published due to the new and growing nature of the field was captured. As pharmacy regulatory bodies are concerned with both pharmacy practice and public safety, we searched the websites of seven pharmacy organizations: International Pharmaceutical Federation, Canadian Pharmacists Association, Canadian Society of Health-Systems Pharmacy, National Association of Pharmacy Regulatory Authorities, American Pharmacists Association, American Society of Health-System Pharmacists, and European Association of Hospital Pharmacists. A keyword search for ‘artificial intelligence’ was performed on each website, and reports were evaluated for eligibility.

Data was first extracted using Covidence to discern what was discussed in papers pertaining to the risks to patients when using AI in pharmacy practice. Studies were categorized as having (1) a few words or sentences detailing risks to patients, (2) an entire section dedicated to discussing risks to patients, or (3) the entire paper was focused on discussion of risk. Then, papers, including the relevant grey literature, were further charted based on citation information, the artificial intelligence tool identified, and details of the reported risk(s) to patients. Data was qualitatively analyzed using general inductive methods and descriptive codes were created to characterize the reported risks to patients. This was done independently by TZ and AP and where discrepancies occurred, a discussion was had until consensus was reached.

## 3. Results

**Figure 1:**
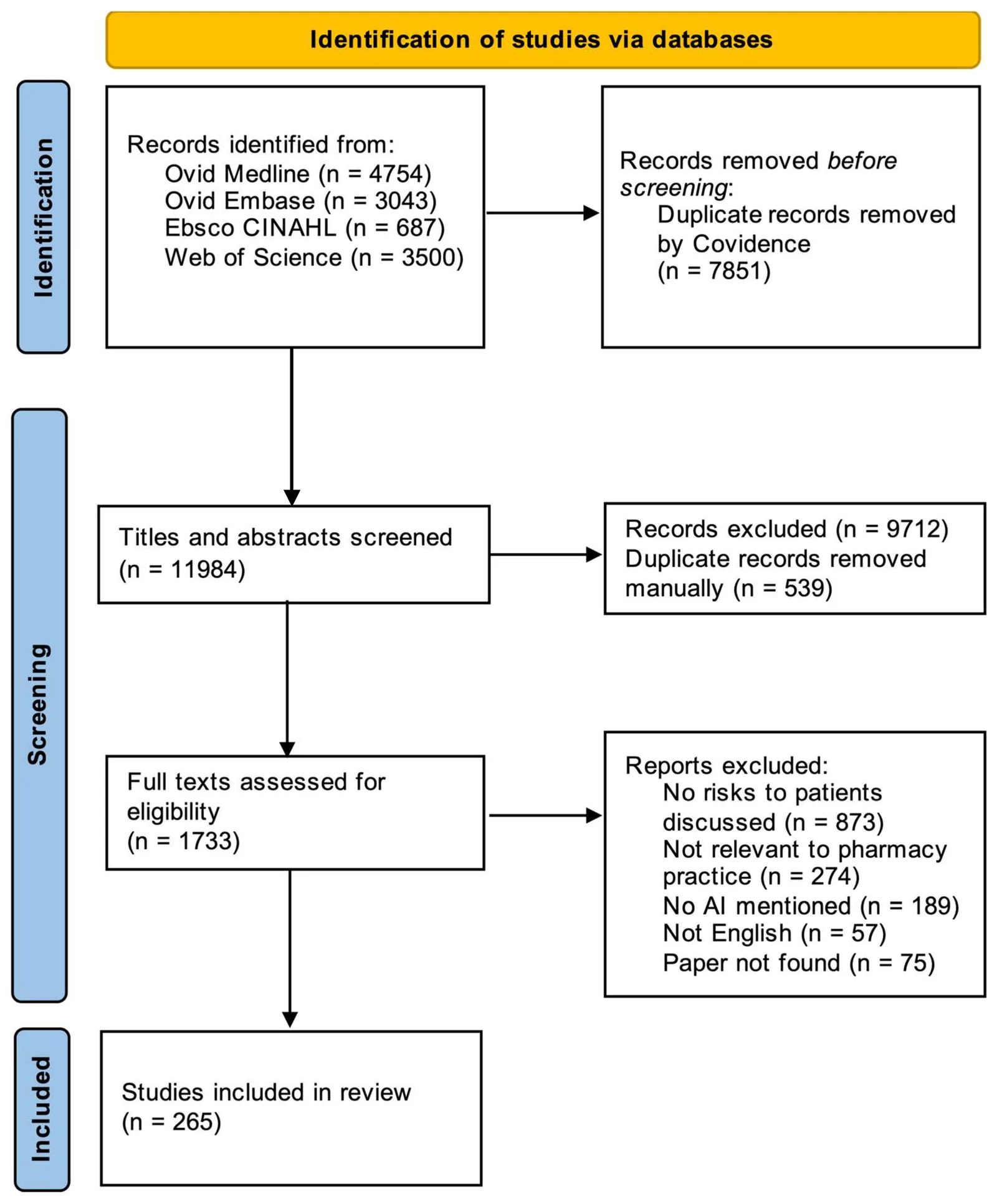
PRISMA flow diagram.

After de-duplication through Covidence, our search resulted in 11,984 papers related to ‘artificial intelligence’ and ‘pharmacy practice’. After title and abstract screening, 9712 papers were excluded as they were either not related to pharmacy practice, with many focused on drug discovery or the pharmaceutical industry. Others excluded at this point did not discuss AI, with many papers focused on simple automation tools such as a pill counter. In addition, 539 duplicates were identified and manually removed. A total of 1733 papers were screened in full text review to confirm the paper pertained to pharmacy practice, discussed an AI tool or system, and reported potential or actual risks to patients. Of the 1468 papers that did not meet the eligibility criteria, 873 did not discuss any risks to patients, 274 were not relevant to pharmacy practice, and 189 did not discuss an AI tool. Although ‘papers written in English’ was a filter placed on our database search, there were 57 papers published in a language other than English that passed through the filter and were excluded. There were 75 papers whose full text could not be found or accessed by study authors with most of these being conference papers and abstracts. Details on the identification of studies can be found in Figure 1. Inter-rater reliability showed strong agreement between authors with proportionate agreement and Cohen’s kappa calculated at 95% and 0.80 for title and abstract screening, respectively, and 95% and 0.79 for full text review. A grey literature search was also conducted. A total of 72 reports related to AI systems and pharmacy practice were found across the websites of seven pharmacy regulatory organizations, including the Canadian Pharmacists Association, American Pharmacists Association and the International Pharmaceutical Federation. After assessing for eligibility, 55 of these reports did not discuss any risks to patients and researchers were unable to access 10 reports due to limited membership access.

There were 265 published papers and seven grey literature reports that fulfilled our eligibility criteria. In terms of publication year, 51% (n = 139) of these studies were published in 2024, 18% (n = 47) were published in 2023, and 17% (n = 45) were published in 2022 or earlier. Since the search was conducted in March of 2025, only 13% (n = 34) studies were published that year. Of the included studies, the aspects of pharmacy practice that were discussed were heterogenous. They included tasks such as answering drug information questions, compounding medications, and monitoring drug levels; various medical conditions that patients whom pharmacists care for may have; or had general commentary around pharmacy practice at large, like published position statements by organizations such as the American Society of Hospital Pharmacists [31,32,33,34,35]. In terms of the AI tools or systems mentioned, 42% (n = 110) discussed large language models or chatbots, 12% (n = 32) discussed various types of machine learning tools (including deep learning), and 40% (n = 107) spoke about AI in more general terms, for example, an overview of various AI tools used in cardiology [34].

Included papers discussed risks to patients to varying depths with 68% (n = 181) of papers mentioning just a few words and not more than two sentences discussing risks, 25% (n = 66) having a dedicated section with at least a paragraph dedicated to describing risks, and 7% (n = 18) written with a larger focus on risk, which study authors defined quantitatively as having as least 50% of the paper discussing risk. Many studies outlined multiple risks rather than focusing solely on one. Proportionate agreement between researchers at this stage was also strong at 98%. Regardless of the depth of discussion on risk, there were three main risk areas identified by the included papers: (1) inaccuracies or the provision of incorrect information to patients, (2) concerns with data security and maintaining privacy of personal health information, and (3) risks associated with the amplification of biases that are present in health data.

### 3.1. Inaccuracies or the Provision of Incorrect Information to Patients

Over 70% (n = 196) of the included studies discussed the direct risk of harm to a patient due to inaccuracies present within various AI outputs. Of the 196 papers that discussed risks associated with inaccuracies, there were varying types of AI tools that were discussed (Table 2). Inaccuracies listed were dependent on the type of AI featured in a paper with two predominant sources of inaccuracies: hallucinations and data-related inaccuracies. Studies also highlighted the importance of having human oversight to mitigate the specific risk of inaccuracies, be they from hallucinations or data-related sources.

#### 3.1.1. AI Hallucinations

As LLMs represented the AI tool of focus for over 50% of the papers listing inaccuracies, AI hallucinations, or the propensity of a chatbot to completely fabricate an output, were often attributed as a mechanism through which inaccuracies appear. Most often, papers superficially mentioned “the risk of LLM hallucinations” [36], that “LLMs […] are prone to disseminating misinformation and exhibiting hallucinations” [37], or that “large language models like ChatGPT are susceptible to hallucinations, or fabrication of information” rather than go into depth examining the specific harms that could come to patients resulting from a hallucination [38]. In addition to papers that focused on LLMs, around 20% (n = 14) of papers that spoke about AI more generally also listed the risk of AI hallucinations contributing to inaccuracies that may pose harms to patients, also mentioning this risk in superficial ways.

#### 3.1.2. Data-Related Limitations

Other studies discussed the propensity for AI systems to output inaccurate information given the limitations of the data used by AI systems. Many of these studies focused on examining ML tools. For example, in a study looking at an algorithm used to optimize depression treatment, authors note that “it would be contraindicated to recommend the current models for clinical use… as the predictions… suffer from poor accuracy and may incur a potential risk of misclassification” [39]. Despite this study leveraging data from the largest clinical trial of second-step depression treatments, the authors recommend utilizing other data such as genetic factors and electronic health record data in order to improve accuracy.

Bucker et al., in discussing AI tools used for clinical decision support, point out limitations related to data that an AI tool may or may not have access to in a given clinical setting, thus impacting its accuracy. “For instance, blood pressure readings while on an ACE inhibitor treatment may vary, with some patients exhibiting normal levels and others experiencing elevated readings. This distinction is challenging for AI to discern, as the model lacks access to the patient’s baseline blood pressure prior to drug intervention, a common limitation in clinical settings” [40].

#### 3.1.3. Importance of Human Oversight

Finally, studies point to the direct harms that may befall patients when inaccurate information is used in a healthcare context, especially when there is no oversight being provided by a healthcare professional [41]. Human oversight is essential to reduce harms associated with AI hallucinations. There is also a close relationship between the type and quality of the data used to train AI systems and the accuracy of the outputs of that system, and there are many existing challenges with the data itself. In a commentary paper, Kleebayoon states that “[it is] impossible to use AI systems of drug knowledge as long as there are obstacles like inaccurate content, missing references and repeatability” [42]. A human-in-the-loop is likely needed to understand the limitations of these systems and reduce the potential harms associated with these inaccuracies.

### 3.2. Data Security and Privacy Concerns

Nearly 50% (n = 121) of included studies listed concerns with data security, discussing the general need to maintain privacy when it comes to patients’ personal health information.

#### 3.2.1. Superficial Discussion of Data Security and Privacy Concerns

While mentioned often, many of these studies presented these concerns in superficial ways, often merely listing “data security and privacy concerns” in superficial ways, as if in passing. Ahn et al. discuss that “the processing of protected health information using [generative] AI raises substantial privacy and security concerns” without further detail of the harms these concerns may pose [43]. Samajdar et al. state “ensuring data privacy and security are a major concern” followed simply by “robust data protection measures are needed to safeguard patient information” [44]. And Zawiah et al. outline concerns of PharmD students regarding “potential breaches in patients’ privacy and confidentiality” in a broad sense without further mention of the types of potential breaches [45].

#### 3.2.2. Ethical Concerns Related to Data Security and Privacy

Some papers move beyond the superficial mention of data and privacy risks by linking these with ethical risks associated with inappropriate or potentially nefarious use of personal health data without consent. Shin et al. state that “the use of ChatGPT in pharmacy presents data security risks because private health information protected by law could be inadvertently entered into the web interface” [46]. Similarly, Nwosu et al. point out an “increased threat to data privacy and protection as robots are likely to access, record and generate a large amount of personal data which could be used without the consent of the individual” [47]. Hasan et al. discuss ethical concerns when it comes to “commercial access to AI outcomes”, calling for transparent definitions of “[data] ownership, access, sharing, and … monitoring to guarantee privacy protection.” [48] Papers also reference existing governance around protection of personal health information and the need to adhere to these laws, such as Crilly’s account that “collecting patient data is governed by General Data Protection Regulation (GDPR) rules in Europe… [and] given the potential risks of malicious hacking, it is important that the pharmacy industry prioritizes its data security” [49].

### 3.3. Risks Associated with Amplifying Biases

Finally, 45% (n = 120) of included studies cited concerns with bias. Similar to risks associated with data security and privacy above, some studies merely mention that there are risks associated with algorithmic bias without a deeper discussion of what these biases entail or what the specific risks may be. Lima et al. point out that there is “potential bias of the information generated by ChatGPT” [50] and Rammal et al. state “ethical considerations… include addressing algorithmic bias” [51]. Other studies described either system-level biases (i.e., existing biases inherent in society and existing data) or biases related to a specific tool.

#### 3.3.1. System-Level, or ‘Algorithmic’ Biases

Studies acknowledged that existing healthcare data is biassed, and that using this data to train AI systems may amplify these biases. Huang et al. state that “bias risks are present since AI algorithms are often trained on biassed datasets. OpenAI even acknowledges that ChatGPT’s output can perpetuate sexist stereotypes” [52]. There is some debate as to the extent of amplification of existing societal biases within AI. In an exploration of whether various AI chatbots exhibited racial/ethnic or sex bias in recommending treatments for pain, Young et al. determined that GPT4 and Gemini did not display any racial/ethnic or sex bias. However, they also acknowledged previous research that did demonstrate that AI chatbots provide different recommendations for pain treatments based on a patient’s race and sex [53].

#### 3.3.2. Biases Related to a Specific Tool

Studies also acknowledged that the data used to train a specific AI tool may cause biases that limit the contexts in which that tool may be used responsibly. Bhargava et al. point out that “training algorithms often require large data sets. In pediatrics, the small sample sizes of some data sets, especially when split by age group, can pose challenges when building unbiassed AI algorithms. Relatedly, in order to build less biased algorithms, training data must be representative of the applicable populations across domain.” [54]. The issue of representation in data leading to potential biases is echoed in a point raised by Wong et al. in their 2023 narrative review of AI in pharmacy practice: “one notable challenge that we highlighted within our review is the evaluation of underrepresented populations, which may affect the generalizability of AI algorithms to these populations” [55].

While the three risk areas listed above constituted the majority of risks listed in the included studies, other types of risks were mentioned, including a lack of transparency of models (10%, n = 28), negative impact on the patient–provider relationship (6%, n = 16), limited accessibility or perpetuation of existing health access disparities (4%, n = 10), lack of reproducibility (3%, n = 9), potentially outdated information (1.5%, n = 4), and sycophantic tendencies (0.4%, n = 1). In the grey literature, there was a similar focus on discussion of the three risk areas outlined above (inaccuracies, data security, and bias). Full data charting of both published papers and grey literature reports is available in the Supplementary Materials.

## 4. Discussion

This scoping review aimed to provide an overview of the literature concerning the reported risks to patients associated with using artificial intelligence tools in pharmacy practice. Of 1733 papers reviewed, only 265 had any discussion of risks to patients. It is important to note that the discussion of risk in this literature was in the realm of theoretical or potential risks to patients, rather than details of actual harms that befell individuals. Of these 265 papers, only 84 had more than a few words or sentences dedicated to discussing risk. The risks that were outlined by these 265 papers fell within three main areas: the risk of AI systems producing inaccurate results, concerns with data security and privacy, and the risk of biases that may exist in existing data being amplified through AI systems.

There has been a call for professional regulatory bodies to provide guidance to pharmacists and other healthcare professionals to ensure both the safeguarding of patients as well as the most responsible adoption of AI within clinical practice [6,23,24]. Regulations are traditionally built on an understanding of risk, empirically evaluating them and creating guardrails against them [25]. This scoping review has highlighted three areas of potential risk associated with using AI in pharmacy practice that may lead to harm to patients. This could be a starting place for pharmacy regulatory bodies in developing corresponding safeguards for AI use—ensuring oversight on the accuracy of AI outputs or mandating data security measures, for example. Similar potential risks have also been identified by other clinician groups such as nurses, which makes sense given the concern for all clinicians towards patient safety [56]. However, this scoping review also showcases the great imbalance that exists in the pharmacy literature when it comes to discussion of the potential benefits or use-cases of AI in pharmacy practice compared to the discussion of the potential risks or harms. While risks concerning inaccuracies, data security and bias are important to understand, there are likely many more risks associated with this powerful technology that are either not discussed at all in the current literature or only appear in a small subset of papers.

Although mentioned in one paper included in this review, could the sycophantic nature of chatbots lead clinicians to believe a wrong recommendation is right, for example? How might this contribute to the potential deskilling of professionals about which many professional regulators are concerned [2]? How might sycophancy also amplify potential biases and existing personal beliefs that clinicians hold, and how might this translate to unintended, or even intended and nefarious harms to patients? As some AI tools such as early warning systems for patient deterioration have reduced mortality rates in hospitals, are there risks when this technology is not used [5]? There are also concerns from other healthcare professional groups that may impact pharmacy practice as well. For example, dietetic professionals have expressed concerns around ‘AI slop’, or the perpetuation of low-quality, oftentimes misleading content that occurs when AI systems are trained on its own low-quality outputs [57]. Additionally, there are already concerns that exist around ‘alert fatigue’ and automation bias with clinical decision support tools that do not use AI or have an AI component. Although not all AI tools may generate an alert or be used in a clinical decision support context, it is surprising that these existing risks associated with digital tools were missing in the AI literature [20,21,22].

This scoping review also highlights that much of the current discussion around risks associated with AI revolves around LLMs or generative AI, specifically. This may be due to the timing of our search and the ‘disruptive’ release of ChatGPT by OpenAI in November 2022. The most-cited risk found through this review was to do with the potential for AI to ‘hallucinate’ a complete falsehood. AI hallucinations are a phenomenon directly associated with large language models, but there are myriads of other technologies that fall under the AI umbrella that may output inaccurate information in different ways. For example, machine learning algorithms, used often in helping pharmacists predict the best dose of medication for a patient, are vulnerable to overfitting to a particular dataset, meaning they may not be able to give an accurate prediction for a patient whose data it has not encountered in training [58]. It is important to note that AI systems other than LLMs exist and are used in pharmacy practice, and it is equally important to understand and study the specific risks associated with each of these tools.

Given the rapid evolution of AI, the heterogeneity of technologies that fall under its umbrella, and the varied ways in which these technologies may be used, it may be impossible to study and characterize every risk associated with every tool and every use case. While this does not make the study of risks associated with AI an unworthy endeavour, it may be impractical for professional regulatory bodies to wait until all of these risks are characterized before developing regulatory safeguards. In addition to utilizing the existing literature and building upon it, there may also be an opportunity to take a different approach to studying risk itself, which may lead to different approaches to safeguarding patients. There is a social component to risk where individuals or groups may have different thoughts and feelings on what is or what is not ‘risky’. Risk perception and risk tolerance are often influenced by an individual’s prior experiences and knowledge, as well as the institutional values and professional identities they embody [59,60]. For example, the relatively slow rate of adoption of AI solutions in healthcare compared to other industries may point to the risk-averse nature of healthcare providers and pharmacists, aligning with the potentially dire impacts their decisions could have on another human’s health and wellbeing. However, given the reported benefits of AI tools—some of which can potentially save lives—there is also a risk to patients associated with not using these tools. Realizing the influence of education and experience on risk perception, perhaps there is also a role for ensuring AI literacy and education amongst clinicians and students.

Given the novelty of AI and the breadth of technologies it encapsulates, there is an urgent need to further study the risks associated with using this technology alongside the study of its benefits. Without this balance and the recognition of the gaps in our knowledge related to AI risks, one can speculate on a parallel regulatory trajectory between AI and social media. The development of social media has been allowed to flourish over the past decade, largely regulation-free, and it has exhibited real risk, causing personal harms to mental health, as well as increasing political polarization amongst populations [61,62]. Only now are governments hoping to place regulations around the safe use of social media. However, this has proven to be a massively difficult feat [63]. With a technology as powerful and now as accessible as AI, there is an urgent need for regulators to act immediately, before the field grows, like social media, ubiquitously embedded within society [6].

There are some strengths and limitations with this scoping review. Although we aimed to be comprehensive, the rapidly growing number of publications in this field and the often-vague descriptions of tools’ mechanisms mean that there is likely research, both published and unpublished, that may not be included in this review. We acknowledge that from the time our search was initially conducted in 2025 to the time of this publication, the conversation related to risks associated with AI use has developed, evidenced by the more recent publications of some of the references used in our discussion above. Despite our best intentions, the volume of publications has made it difficult to feasibly update our search. Our team of 1.5 full time equivalent (FTE) researchers took one year to screen over 10,000 titles and abstracts and then over 1700 full texts. This is consistent with reported lengths of time required for screening the literature for other knowledge syntheses [64]. An updated search reveals a doubling of publications related to AI and pharmacy practice in the last year alone. With this rapid expansion of the literature, we may always experience some latency in publication time with work in this field; however, authors felt that sharing the results of this work was still important in order to detail a ‘state of affairs’ related to risk within the pharmacy literature at this early stage of AI growth and adoption within pharmacy practice. Additionally, we acknowledge that our review only included the English language literature and our grey literature search focused on pharmacy organizations mainly in North America. As such, our results may not be generalized to pharmacy practice worldwide. Reported risks are greatly influenced by the healthcare systems and local contexts, and we acknowledge that a North American pharmacy context and associated risks are not universal. We also recognize that the regulatory environment is different between Canada, the USA and Europe, especially pertaining to AI, with Europe employing generally more stringent regulations on this technology through the European Union AI Act compared with a general lack of similarly stringent regulations in the American context. Thus, our commentary around regulatory action may apply differently to these varied contexts. A strength of this study was the involvement of pharmacist researchers. This allowed for nuance in the selection of a broad range of studies that are relevant to pharmacists and pharmacy practice. This study focused on pharmacy practice, but many of the included studies discussed medical conditions or body systems more generally. Given the similar knowledge and training that pharmacists, nurses, physicians, and other healthcare professionals possess, there may be insights from this study that could apply to other healthcare professions as well.

## 5. Conclusions

This scoping review has highlighted the large gap in the literature that exists around discussing and characterizing the specific risks to patients associated with using various artificial intelligence tools in pharmacy practice. Artificial intelligence is and will continue to bring important innovation to our healthcare system in order to support ageing, chronically ill populations amidst increasing difficulties in accessing care. However, there is a need to better understand the risks to patients associated with using these technologies in clinical practice so that harms may be prevented before they reach patients. This scoping review brought forward from the literature three commonly reported potential risks to patients associated with using AI in pharmacy practice from the literature. While this may be a starting point for regulators to develop safeguards, characterizing and safeguarding against every single risk associated with AI use is likely an impossible task given the fast-evolving nature of the field.

## 6. Future Directions

Moving forward, taking a different approach to studying and understanding risk and how various key interest groups perceive and tolerate risks in this field may be warranted. This work should be undertaken urgently, and regulatory bodies must act now to implement necessary guardrails, while being careful not to stifle innovation, before these tools become so ingrained in society and much more difficult to regulate.

## Figures and Tables

**Table 1:** Eligibility criteria.

Inclusion Criteria	Exclusion Criteria
Any discussion or identification of negative impacts of AI to patients (any person seeking or receiving healthcare) including risks inherent in technical aspects of the AI (i.e., within the code or data set used), physical or psychological risks to patients, perceived or actual risks of above	Use of AI in the pharmaceutical industry, drug discovery, or research and development related to drugs
Pharmacy practice in all settings where patient care is provided (e.g., community, primary care, hospital, remote settings)	Negative impacts of AI are not explicitly identified or discussed by authors
All types of AI including human-in-loop and human-out-of-loop	Referring to technology that is not AI
Any AI tool used by pharmacists to complete an activity or provide a service that directly impacts a patient	Studies for which an English translation cannot be found

**Table 2:** Breakdown of type of AI tool discussed in papers that mention inaccuracies as a risk.

AI Tool	% of All Papers That Mention Inaccuracies (n = 196)
Large language model (LLM)	52% (n = 101)
Machine learning (ML) tool	11% (n = 21)
General discussion of AI (i.e., AI in cardiology)	33% (n = 64)
Other (computer vision, robotics, AI used for clinical decision support)	5% (n = 10)

## Data Availability

The original contributions presented in this study are included in the article/Supplementary Materials. Further inquiries can be directed to the corresponding authors.

## Supplementary Materials

The following supporting information can be downloaded at: https://www.mdpi.com/article/10.3390/pharmacy14050113/s1, Table S1. Preferred Reporting Items for Systematic reviews and Meta-Analyses extension for Scoping Reviews (PRISMA-ScR) Checklist, Table S2: Data charting for published papers; Table S3: Data charting for grey literature reports; Figure S1. PRISMA 2020 flow diagram.

## Abbreviations

The following abbreviations are used in this manuscript:

AI	Artificial intelligence
LLM	Large language model

## Appendix A

**Table A1:** Ovid Medline search strategy.

#	Searches	Results
1	exp Artificial Intelligence/	228,579
2	((artificial* or machin* or comput*) adj3 intelligen*).ti,ab,kf.	74,314
3	AI.ti,ab,kf.	72,233
4	((predict* or generat*) adj3 model*).ti,ab,kf	264,559
5	(machin* adj3 learn*).ti,ab,kf	147,284
6	natural language process*.ti,ab,kf	11,817
7	((language* learn* or large language*) adj3 model*).ti,ab,kf	5270
8	(generat* adj3 transform*).ti,ab,kf	4126
9	neural network*.ti,ab,kf	129,910
10	((intelligen* or expert*) adj3 (automat* or system*)).ti,ab,kf	16,785
11	(human* adj3 (in or out) adj3 loop*).ti,ab,kf	1177
12	cognitive comput*.ti,ab,kf	323
13	automat*reason*.ti,ab,kf	163
14	deep learn*.ti,ab,kf	83,300
15	(smart adj3 technolog*).ti,ab,kf	2409
16	(Bing chat or ChatGPT* or Chat GPT or Google* Bard or Google* Gemini or IBM Watson or Microsoft* Bing or Microsoft* Copilot or OpenAI, or Open AI or PathAI or Path AI or Perplexity or Claude or Midjourney or DALL-E or DeepAI or Meta* Llama).ti,ab,kf	8551
17	pharmaceutical services/ or community pharmacy services/ or exp drug information services/ or medication therapy management/ or pharmaceutical services, online/ or pharmacy service, hospital/	44,879
18	((clinic* or hospital or community or retail or primary or ambulatory or remote* or tele* or online) adj3 pharmac*).ti,ab,kf	68,811
19	(medication* adj3 (manag* or adher*)).ti,ab,kf	35,403
20	((drug or medication*) adj3 info*).ti,ab,kf	15,743
21	(pharmac* adj3 (prescri* or order* or care* or intervention* or practice* or educat* or student*)).ti,ab,kf	75,258
22	pharmacovigilan*.ti,ab,kf	9584
23	1 or 2 or 3 or 4 or 5 or 6 or 7 or 8 or 9 or 10 or 11 or 12 or 13 or 14 or 15 or 16	683,203
24	17 or 18 or 19 or 20 or 21 or 22	204,901
25	23 and 24	4787
