# Supplementary material for: The Risks to Patients Associated with Using Artificial Intelligence Tools in Pharmacy Practice: A Scoping Review
Source: Pharmacy (Basel). 2026 Aug 3;14(5):113. doi: 10.3390/pharmacy14050113 (PMC13516801; doi:10.3390/pharmacy14050113)
Supplement: Supplementary file 1 [file pharmacy-14-00113-s001.zip › pharmacy-4427750-supplementary.pdf]

Table S1. Preferred Reporting Items for Systematic reviews and Meta-Analyses extension for Scoping Reviews (PRISMA-ScR) Checklist

SECTION	ITEM	PRISMA-ScR CHECKLIST ITEM	REPORTED ON PAGE #
**TITLE**			
Title	1	Identify the report as a scoping review.	1
**ABSTRACT**			
Structured summary	2	Provide a structured summary that includes (as applicable): background, objectives, eligibility criteria, sources of evidence, charting methods, results, and conclusions that relate to the review questions and objectives.	1
**INTRODUCTION**			
Rationale	3	Describe the rationale for the review in the context of what is already known. Explain why the review questions/objectives lend themselves to a scoping review approach.	1-3
Objectives	4	Provide an explicit statement of the questions and objectives being addressed with reference to their key elements (e.g., population or participants, concepts, and context) or other relevant key elements used to conceptualize the review questions and/or objectives.	3
**METHODS**			
Protocol and registration	5	Indicate whether a review protocol exists; state if and where it can be accessed (e.g., a Web address); and if available, provide registration information, including the registration number.	3
Eligibility criteria	6	Specify characteristics of the sources of evidence used as eligibility criteria (e.g., years considered, language, and publication status), and provide a rationale.	3
Information sources*	7	Describe all information sources in the search (e.g., databases with dates of coverage and contact with authors to identify additional sources), as well as the date the most recent search was executed.	3, 4
Search	8	Present the full electronic search strategy for at least 1 database, including any limits used, such that it could be repeated.	10
Selection of sources of evidence†	9	State the process for selecting sources of evidence (i.e., screening and eligibility) included in the scoping review.	3, 4
Data charting process‡	10	Describe the methods of charting data from the included sources of evidence (e.g., calibrated forms or forms that have been tested by the team before	4

SECTION	ITEM	PRISMA-ScR CHECKLIST ITEM	REPORTED ON PAGE #
		their use, and whether data charting was done independently or in duplicate) and any processes for obtaining and confirming data from investigators.	
Data items	11	List and define all variables for which data were sought and any assumptions and simplifications made.	3, 4
Critical appraisal of individual sources of evidence§	12	If done, provide a rationale for conducting a critical appraisal of included sources of evidence; describe the methods used and how this information was used in any data synthesis (if appropriate).	N/A
Synthesis of results	13	Describe the methods of handling and summarizing the data that were charted.	4
**RESULTS**			
Selection of sources of evidence	14	Give numbers of sources of evidence screened, assessed for eligibility, and included in the review, with reasons for exclusions at each stage, ideally using a flow diagram.	4, 5
Characteristics of sources of evidence	15	For each source of evidence, present characteristics for which data were charted and provide the citations.	4, 5
Critical appraisal within sources of evidence	16	If done, present data on critical appraisal of included sources of evidence (see item 12).	N/A
Results of individual sources of evidence	17	For each included source of evidence, present the relevant data that were charted that relate to the review questions and objectives.	Supplementary materials, tables S1 and S2
Synthesis of results	18	Summarize and/or present the charting results as they relate to the review questions and objectives.	6, 7
**DISCUSSION**			
Summary of evidence	19	Summarize the main results (including an overview of concepts, themes, and types of evidence available), link to the review questions and objectives, and consider the relevance to key groups.	7-9
Limitations	20	Discuss the limitations of the scoping review process.	9
Conclusions	21	Provide a general interpretation of the results with respect to the review questions and objectives, as well as potential implications and/or next steps.	9
**FUNDING**			
Funding	22	Describe sources of funding for the included sources of evidence, as well as sources of funding for the scoping review. Describe the role of the funders of the scoping review.	9, 10

JBI = Joanna Briggs Institute; PRISMA-ScR = Preferred Reporting Items for Systematic reviews and Meta-Analyses extension for Scoping Reviews.

* Where *sources of evidence* (see second footnote) are compiled from, such as bibliographic databases, social media platforms, and Web sites.
† A more inclusive/heterogeneous term used to account for the different types of evidence or data sources (e.g., quantitative and/or qualitative research, expert opinion, and policy documents) that may be eligible in a scoping review as opposed to only studies. This is not to be confused with *information sources* (see first footnote).
‡ The frameworks by Arksey and O'Malley (6) and Levac and colleagues (7) and the JBI guidance (4, 5) refer to the process of data extraction in a scoping review as data charting.
§ The process of systematically examining research evidence to assess its validity, results, and relevance before using it to inform a decision. This term is used for items 12 and 19 instead of "risk of bias" (which is more applicable to systematic reviews of interventions) to include and acknowledge the various sources of evidence that may be used in a scoping review (e.g., quantitative and/or qualitative research, expert opinion, and policy document).

From: Tricco AC, Lillie E, Zarin W, O'Brien KK, Colquhoun H, Levac D, et al. PRISMA Extension for Scoping Reviews (PRISMAScR): Checklist and Explanation. Ann Intern Med. 2018;169:467–473. doi: 10.7326/M18-0850.

Figure S1. PRISMA 2020 flow diagram

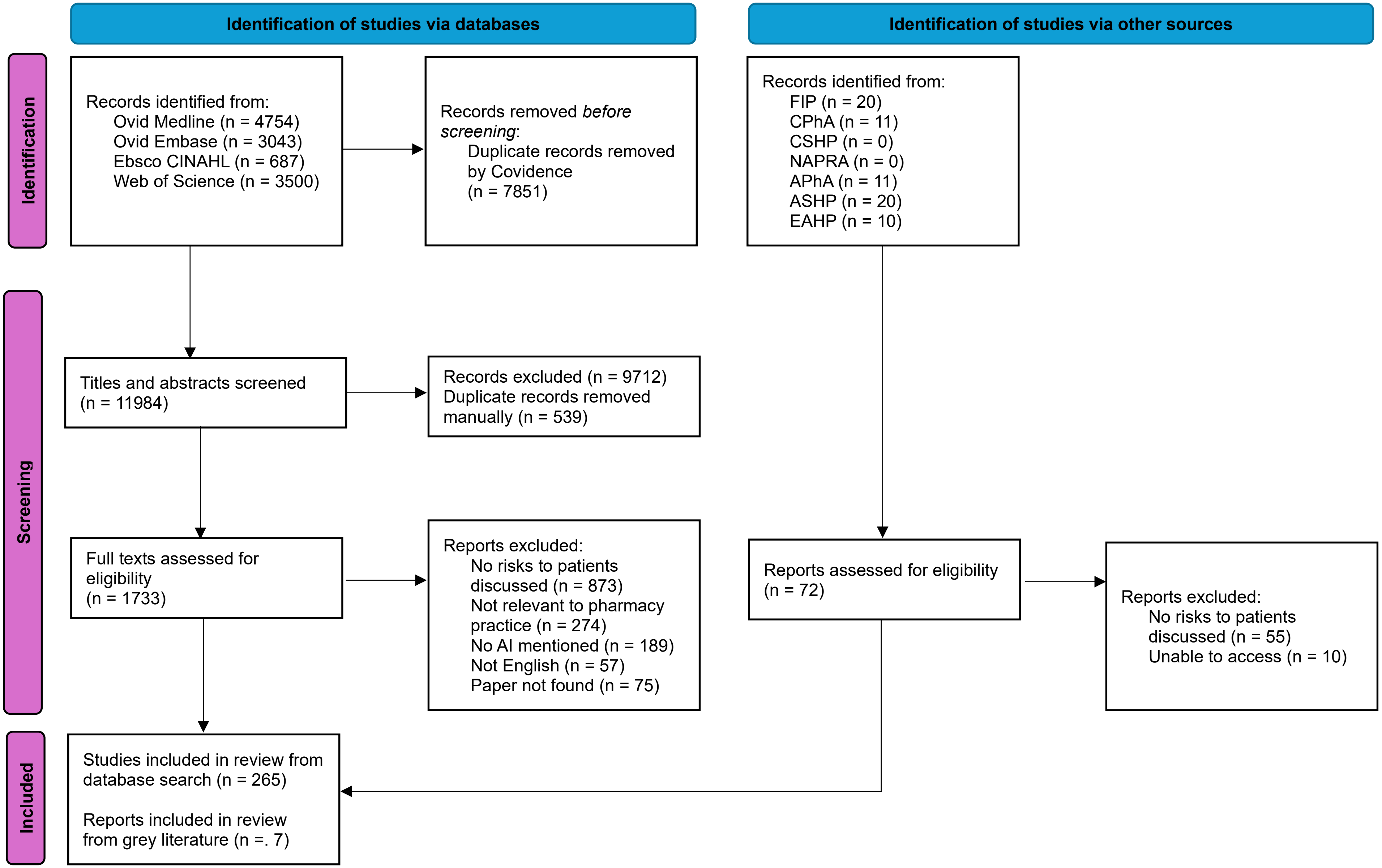

Table S2: Data Charting for Published Papers

					Risks described								
**Year**	**Study ID**	**Title**	**AI tool/ system investigated**	**Extent of risks reported**	**Bias**	**Privacy/Security**	**Inaccuracies**	**Transparency (Black Box)**	**Reproducibility**	**Accessibility**	**Sycophancy**	**Outdated**	**Patient-Provider Relationship**
**2024**	Abavisani 2024	Chatting with artificial intelligence to combat antibiotic resistance: Opportunities and challenges	LLM	A dedicated section	Y	Y	Y			Y			Y
**2024**	AbdelAziz 2024	A scoping review of artificial intelligence within pharmacy education.	General	A few words or sentences		Y	Y						
**2024**	Abid 2024	Breaking Barriers in Behavioral Change: The Potential of Artificial Intelligence-Driven Motivational Interviewing.	LLM	A dedicated section	Y	Y	Y						
**2023**	Abu-Farha 2023	Evaluation of community pharmacists' perceptions and willingness to integrate ChatGPT into their pharmacy practice: A study from Jordan.	LLM	A dedicated section	Y	Y	Y						Y
**2023**	AbuHammour 2023	ChatGPT in pharmacy practice: a cross-sectional exploration of Jordanian pharmacists' perception, practice, and concerns.	LLM	A dedicated section	Y	Y	Y						
**2023**	ACM 2023	Unpicking Epistemic Injustices in Digital Health: On the Implications of Designing Data-Driven Technologies for the Management of Long-Term Conditions	General	The paper is focused on risk	Y								
**2021**	AFTAB 2021	Classification of Failures in the Perception of Conversational Agents (CAs) and Their Implications on Patient Safety...Medical Informatics Europe, Public Health and Informatics Conference (Virtual), 29-31 May, 2021.	LLM	The paper is focused on risk			Y						
**2021**	Agarwal 2021	Artificial intelligence in the pharmacy profession	General	A dedicated section		Y	Y						
**2022**	Aggarwal 2022	Artificial Intelligence (AI)-based Chatbots in Promoting Health Behavioral Changes: A Systematic Review	LLM	A dedicated section		Y	Y						
**2024**	Aguero 2024	The Potential Application of Large Language Models in Pharmaceutical Supply Chain Management	LLM	A dedicated section		Y	Y						
**2025**	Ahn 2025	Application of Generative Artificial Intelligence in Dyslipidemia Care.	LLM	A few words or sentences		Y	Y					Y	
**2024**	Akamine 2024	Effects of temperature settings on information quality of ChatGPT-3.5 responses: A prospective, single-blind, observational cohort study	LLM	A few words or sentences			Y						
**2023**	Al-Dujaili 2023	Assessing the accuracy and consistency of ChatGPT in clinical pharmacy management: A preliminary analysis with clinical pharmacy experts worldwide.	LLM	A few words or sentences			Y		Y				
**2024**	Alabdulqader 2024	Empowering Home Care: Utilizing IoT and Deep Learning for Intelligent Monitoring and Management of Chronic Diseases	ML	A dedicated section		Y							
**2024**	Alahmadi 2024	A Computer Vision-Based Pill Recognition Application: Bridging Gaps in Medication Understanding for the Elderly	Computer vision	A few words or sentences			Y						

**2022**	Alahmari 2022	An Overview of the Current State and Perspectives of Pharmacy Robot and Medication Dispensing Technology.	General	A few words or sentences			Y						
**2024**	Albogami 2024	Safety and quality of AI chatbots for drug-related inquiries: A real-world comparison with licensed pharmacists.	LLM	A dedicated section			Y						
**2024**	Alkanj 2024	Deep learning classification of drug-related problems from pharmaceutical interventions issued by hospital clinical pharmacists during medication prescription review: a large-scale descriptive retrospective study in a French university hospital.	ML	A few words or sentences	Y								
**2025**	Allam 2025	Prescribing the Future: The Role of Artificial Intelligence in Pharmacy	General	A dedicated section	Y	Y	Y						
**2024**	Almeman 2024	The digital transformation in pharmacy: embracing online platforms and the cosmeceutical paradigm shift.	General	A few words or sentences									
**2024**	Alsanosi 2024	Potential Applications of Artificial Intelligence (AI) in Managing Polypharmacy in Saudi Arabia: A Narrative Review.	General	A few words or sentences		Y							
**2021**	Altamura 2021	Algorithms and Artificial Intelligence in Primary Care for conscious drug use: a systematic review...14th European Public Health Conference (Virtual), Public health futures in a changing world, November 10-12, 2021	General	A few words or sentences			Y						
**2024**	Altschuler 2024	Clinician as editor: notes in the era of AI scribes	LLM	A few words or sentences	Y		Y						
**2024**	Anderson 2024	Pharmacy student use of ChatGPT: A survey of students at a U.S. School of Pharmacy.	LLM	A few words or sentences			Y						
**2025**	Andrikyan 2025	Artificial intelligence-powered chatbots in search engines: a cross-sectional study on the quality and risks of drug information for patients.	LLM	The paper is focused on risk			Y						
**2023**	Angel 2023	Clinical Knowledge and Reasoning Abilities of AI Large Language Models in Pharmacy: A Comparative Study on the NAPLEX Exam	LLM	A few words or sentences			Y						
**2025**	Anonymous 2025	Correction to: Let's Have a Chat: How Well Does an Artificial Intelligence Chatbot Answer Clinical Infectious Diseases Pharmacotherapy Questions?.	LLM	A few words or sentences			Y						
**2025**	Antwerp 2025	Emerging trends in information technology: Perspectives on the 2024 AMCP Foundation Survey	General	A few words or sentences	Y	Y	Y	Y					
**2024**	Armstrong 2024	Can artificial intelligence (AI) educate your patient? A study to assess overall readability and pharmacists' perception of AI-generated patient education materials	LLM	A few words or sentences	Y		Y	Y					
**2022**	Aronson 2022	Artificial Intelligence in Pharmacovigilance: An Introduction to Terms, Concepts, Applications, and Limitations.	General	A dedicated section			Y						
**2025**	Aydin 2025	Navigating the potential and pitfalls of large language models in patient-centered medication	LLM	The paper is focused on risk			Y						

		guidance and self-decision support.											
**2021**	Babel 2021	Artificial Intelligence Solutions to Increase Medication Adherence in Patients With Non-communicable Diseases.	General	A dedicated section	Y	Y							
**2024**	Bakker 2024	Standard of practice in pharmacy informatics	General	A dedicated section	Y								
**2024**	Balendran 2024	<i>Algorithmovigilance</i>, lessons from pharmacovigilance	General	The paper is focused on risk	Y		Y	Y					
**2024**	Batson 2024	The Pharmacy Students' Guide to Artificial Intelligence-AI.	General	A few words or sentences	Y		Y						
**2024**	Bazzari 2024	Utilizing ChatGPT in Telepharmacy.	LLM	A few words or sentences		Y	Y						
**2024**	Bazzari 2024	Assessing the ability of GPT-4o to visually recognize medications and provide patient education.	LLM	A few words or sentences			Y						
**2023**	Beavers 2023	Evaluation of inpatient medication guidance from an artificial intelligence chatbot.	LLM	A few words or sentences			Y						
**2024**	Bhargava 2024	Promises, Pitfalls, and Clinical Applications of Artificial Intelligence in Pediatrics	General	A dedicated section	Y					Y			
**2021**	Bohlmann 2021	Machine Learning and Medication Adherence: Scoping Review.	ML	A few words or sentences			Y						
**2024**	Boonrit 2024	Results of the COMPARE-GPT study: Comparison of medication package inserts and GPT-4 cancer drug information	LLM	A few words or sentences			Y						
**2024**	Bora 2024	AI-POWERED HEALTH MONITORING: ENHANCING CHRONIC DISEASE MANAGEMENT	LLM	A few words or sentences	Y	Y	Y						
**2025**	Bouabida 2025	Navigating the landscape of remote patient monitoring in Canada: trends, challenges, and future directions.	General	A few words or sentences	Y	Y	Y	Y					
**2022**	Bu 2022	Artificial intelligence-based internet hospital pharmacy services in China: Perspective based on a case study.	General	A few words or sentences		Y				Y			
**2024**	Bucker 2024	Artificial intelligence to assist decision-making on pharmacotherapy: A feasibility study.	ML	A dedicated section			Y						
**2024**	Campbell 2024	Performance of the ChatGPT large language model for decision support in community pharmacy.	LLM	A few words or sentences		Y							
**2024**	Carini 2024	Tribulations and future opportunities for artificial intelligence in precision medicine.	General	A dedicated section	Y	Y							
**2023**	Cascella 2023	Exploring Artificial Intelligence in Anesthesia: A Primer on Ethics, and Clinical Applications	General	A dedicated section	Y	Y		Y					
**2023**	Chalasani 2023	Artificial intelligence in the field of pharmacy practice: A literature review.	General	A dedicated section	Y	Y							
**2024**	Chen 2024	Ability of machine-learning based clinical decision support system to reduce alert fatigue, wrong-drug errors, and alert users about look alike, sound alike medication.	ML	A few words or sentences									
**2024**	Chinene 2024	Insights into GenAI: Perspectives of radiography and pharmacy	LLM	A few words or sentences	Y	Y	Y						

		students at a leading institution in Zimbabwe											
**2024**	Cho 2024	Application of artificial intelligence in hypertension.	General	A few words or sentences	Y	Y							
**2024**	Clark 2024	Chatbots in Health Care: Connecting Patients to Information: Emerging Health Technologies	LLM	A dedicated section	Y	Y				Y			
**2024**	Cordero-Cruz 2024	Conquering the future in hospital pharmacy: Training as a pillar of success	General	A few words or sentences	Y	Y	Y						
**2024**	Cornelison 2024	Accuracy of a chatbot in answering questions that patients should ask before taking a new medication.	LLM	A few words or sentences			Y						
**2020**	Corrigan 2020	Artificial Intelligence and Machine Learning: Will Clinical Pharmacologists Be Needed in the Next Decade? The John Henry Question.	General	A few words or sentences			Y	Y					
**2020**	Cresswell 2020	Investigating the use of data-driven artificial intelligence in computerised decision support systems for health and social care: A systematic review.	General	A few words or sentences			Y						
**2023**	Crilly 2023	Opportunities and threats for community pharmacy in the era of enhanced technology and artificial intelligence.	General	A few words or sentences		Y							
**2024**	Curtiss 2024	Optimizing precision medicine for second-step depression treatment: a machine learning approach.	ML	A few words or sentences			Y						
**2021**	D 2021	Digital health implications for clinical pharmacists services: A primer on the current landscape and future concerns	General	A few words or sentences		Y	Y						
**2024**	Dachary 2024	Pharmacy Practice Pharmaceutical Technology in Enhancing Healthcare Efficiency and Patient Care	General	A few words or sentences		Y							
**2023**	Damiani 2023	Potentiality of algorithms and artificial intelligence adoption to improve medication management in primary care: a systematic review.	General	A few words or sentences		Y		Y					
**2024**	Dara 2024	Advancing medical imaging: detecting polypharmacy and adverse drug effects with Graph Convolutional Networks (GCN).	GNN	A few words or sentences	Y	Y							
**2024**	Das 2024	Improved precision oncology question-answering using agentic LLM	LLM	A few words or sentences	Y		Y						
**2024**	Daungsupawong 2024	Drug information question responses by a drug information center and by ChatGPT: Correspondence.	LLM	A few words or sentences			Y						
**2025**	deDonato 2025	Development and Content Analysis Protocol for Evaluating Artificial Intelligence in Drug-Related Information.	LLM	A few words or sentences			Y						
**2019**	Dentzer 2019	Creating the future of artificial intelligence in health-system pharmacy.	General	A few words or sentences			Y						
**2025**	deRuiter 2025	The extent and type of use, opportunities and concerns of ChatGPT in community pharmacy: A survey of community pharmacy staff.	LLM	The paper is focused on risk		Y	Y	Y	Y				

**2024**	Devliyal 2024	EiAiMSPS: Edge Inspired Artificial Intelligence-based Multi Stakeholders Personalized Security Mechanism in iCPS for PCS	ML	A dedicated section		Y	Y						
**2025**	Devraj 2025	Bridging the Digital Divide: The Importance of Techquity in Pharmacy Education.	General	The paper is focused on risk	Y								
**2024**	Dickinson 2024	Learning with an evolving medicine label: how artificial intelligence-based medication recommendation systems must adapt to changing medication labels.	ML	A few words or sentences	Y		Y						
**2025**	Edwards 2025	The role of artificial intelligence in emergency medicine pharmacy practice.	General	A dedicated section	Y	Y	Y	Y					
**2020**	Eggerth 2020	Medication management needs information and communications technology-based approaches, including telehealth and artificial intelligence.	General	A few words or sentences		Y							
**2024**	Ehlert 2024	Large Language Models and the North American Pharmacist Licensure Examination (NAPLEX) Practice Questions.	LLM	A few words or sentences			Y						
**2024**	Ernst 2024	Clinical pharmacy and patient safety: Have we reached a golden era?	General	A few words or sentences			Y						
**2024**	Falconer 2024	Powered by AI: advancing towards artificial intelligence algorithms in Australian hospital pharmacy	General	A dedicated section	Y	Y	Y	Y					
**2025**	Feng 2025	Knowledge graph-based thought: a knowledge graph-enhanced LLM framework for pan-cancer question answering	LLM	A few words or sentences			Y						
**2024**	Fiordelisi 2024	ChatGPT, clinical pharmacist's ally in verifying herbal-drug interactions: Potential and limitations	LLM	A few words or sentences		Y	Y						
**2019**	Flynn 2019	Using artificial intelligence in health-system pharmacy practice: Finding new patterns that matter.	General	A dedicated section	Y		Y						
**2024**	Fournier 2024	Assessing the applicability and appropriateness of ChatGPT in answering clinical pharmacy questions.	LLM	A few words or sentences			Y						
**2024**	Gagaille 2024	Qualification and impact of a video-assisted control system in a chemotherapy compounding unit.	Robotics	A few words or sentences			Y						
**2023**	GarciaValencia 2023	Enhancing Kidney Transplant Care through the Integration of Chatbot.	LLM	A dedicated section		Y	Y						Y
**2023**	Gellad 2023	Development and validation of an overdose risk prediction tool using prescription drug monitoring program data.	ML	A few words or sentences	Y		Y						
**2025**	Ghozali 2025	Assessing ChatGPT's accuracy and reliability in asthma general knowledge: implications for artificial intelligence use in public health education.	LLM	A dedicated section	Y	Y	Y						Y
**2024**	Gijsen 2024	Does artificial intelligence close gaps in clinical pharmacology in the ICU?	General	A dedicated section			Y	Y					

**2024**	Goldman 2024	Assessing ChatGPT's impact on curriculum outcomes and Entrustable Professional Activities (EPAs) during Advanced Pharmacy Practice Experiences (APPEs)	LLM	A few words or sentences									
**2024**	Gondode 2024	Comparing patient education tools for chronic pain medications: Artificial intelligence chatbot versus traditional patient information leaflets.	LLM	A few words or sentences	Y		Y						
**2024**	GonzálezMuñoz 2024	Opportunities and Challenges for AI in Long-Term Care: Pharmacists' Perspectives.	General	A few words or sentences	Y	Y	Y						
**2021**	Gregorio 2021	The pharmacist's guide to the future: Are we there yet?.	General	A few words or sentences	Y	Y				Y			
**2025**	Griné 2025	Digital health in cardiovascular medicine: An overview of key applications and clinical impact by the Portuguese Society of Cardiology Study Group on Digital Health	General	A few words or sentences	Y		Y	Y					Y
**2024**	Grossman 2024	Appropriateness of ChatGPT as a resource for medication-related questions.	LLM	A few words or sentences			Y						
**2023**	Guan 2023	Artificial intelligence in diabetes management: Advancements, opportunities, and challenges.	General	A dedicated section	Y	Y	Y						
**2025**	Gustafson 2025	Pharmacists' perceptions of artificial intelligence: A national survey.	General	A few words or sentences	Y	Y	Y						
**2007**	Haddad 2007	Neural network adaptive output feedback control for intensive care unit sedation and intraoperative anesthesia.	CDSS	A few words or sentences			Y						
**2024**	Haga 2024	Artificial intelligence, medications, pharmacogenomics, and ethics	General	A dedicated section	Y	Y	Y	Y					Y
**2023**	Han 2023	MCFF-MTDDI: multi-channel feature fusion for multi-typed drug-drug interaction prediction.	ML	A few words or sentences	Y								
**2025**	Han 2025	Usefulness and limitations of Chat GPT in getting information on teratogenic drugs exposed in pregnancy	LLM	A dedicated section			Y						
**2020**	Hardcastle 2020	Virtual care: Enhancing access or harming care?	General	A dedicated section		Y	Y						
**2022**	Harris 2022	Clinical deployment environments: Five pillars of translational machine learning for health	General	A dedicated section			Y	Y					
**2011**	Hasan 2011	Automatic detection of omissions in medication lists	LLM	A few words or sentences			Y						
**2024**	Hasan 2024	Perspectives of Pharmacy Students on Ethical Issues Related to Artificial Intelligence: A Comprehensive Survey Study.	General	A few words or sentences	Y	Y				Y			
**2024**	Hasan 2024	Ethical considerations and concerns in the implementation of AI in pharmacy practice: a cross-sectional study.	General	A dedicated section	Y	Y				Y			Y
**2022**	Hassan 2022	HEALTHCARE STAFF PERCEPTIONS ON USING ARTIFICIAL INTELLIGENCE PREDICTIVE TOOLS: A QUALITATIVE STUDY	General	A few words or sentences			Y						
**2024**	Hatem 2024	Advancing Pharmacy Practice: The Role of Intelligence-Driven Pharmacy Practice and the	General	A dedicated section	Y	Y							

		Emergence of Pharmacointelligence											
**2023**	Haze 2023	Influence on the accuracy in ChatGPT: Differences in the amount of information per medical field	LLM	A few words or sentences			Y						
**2025**	He 2025	Chat GPT-4 significantly surpasses GPT-3.5 in drug information queries.	LLM	A few words or sentences			Y						
**2024**	Helgestad 2024	ChatGPT versus physician-derived answers to drug-related questions.	LLM	A few words or sentences			Y						
**2023**	Heo 2023	Development and validation of a machine learning-based fall-related injury risk prediction model using nationwide claims database in Korean community-dwelling older population.	ML	A few words or sentences			Y						
**2023**	HOLLAN 2023	Navigating the Data Privacy Minefields in Pharma's Use of AI: Industry professionals weigh in on key learnings to date.	General	A few words or sentences	Y	Y	Y						
**2024**	Hoti 2024	Encouraging dissemination of research on the use of artificial intelligence and related innovative technologies in clinical pharmacy practice and education: call for papers.	General	A few words or sentences	Y	Y	Y						
**2024**	Huang 2024	Evaluating the performance of ChatGPT in clinical pharmacy: A comparative study of ChatGPT and clinical pharmacists.	LLM	A dedicated section			Y						
**2024**	Hundal 2024	Results of the COMPARE-GPT study: Comparison of medication package inserts and GPT-4 cancer drug information	LLM	A few words or sentences			Y						
**2006**	IEEE 2006	Adaptive and neural network adaptive control of depth of anesthesia during surgery	CDSS	A few words or sentences			Y						
**2024**	Ilkic 2024	Prospective systematic risk analysis of the digital technology use within pharmaceutical care.	General	A dedicated section	Y	Y	Y						
**2023**	Iwasawa 2023	Knowledge and attitudes of pharmacy students towards artificial intelligence and the ChatGPT	LLM	A few words or sentences			Y						
**2023**	Jairoun 2023	ChatGPT: Threat or boon to the future of pharmacy practice?.	LLM	A dedicated section		Y	Y						
**2024**	Jairoun 2024	Capturing pharmacists' perspectives on the value, risks, and applications of ChatGPT in pharmacy practice: A qualitative study.	LLM	A dedicated section		Y	Y						Y
**2023**	Janus 2023	A Comparative Analysis of Chatgpt Vs Expert in Managing Anticancer Drug in Patients Renal Insufficiency	LLM	A few words or sentences			Y						
**2023**	Jarab 2023	Artificial intelligence in pharmacy practice: Attitude and willingness of the community pharmacists and the barriers for its implementation.	General	A few words or sentences			Y						
**2023**	Jarab 2023	Artificial intelligence (AI) in pharmacy: an overview of innovations.	General	A dedicated section		Y	Y	Y					
**2024**	Jin 2024	Performance of ChatGPT-3.5 and GPT-4 in national licensing examinations for medicine, pharmacy, dentistry, and nursing: a systematic review and meta-analysis.	LLM	A few words or sentences			Y					Y	

**2024**	Jin 2024	Performance of GPT-3.5 and GPT-4 on the Korean Pharmacist Licensing Examination: Comparison Study.	LLM	A few words or sentences			Y						
**2023**	Johns 2023	USING MACHINE LEARNING OR DEEP LEARNING MODELS IN A HOSPITAL SETTING TO DETECT INAPPROPRIATE PRESCRIPTIONS: A SYSTEMATIC REVIEW	ML	A few words or sentences	Y		Y					Y	
**2020**	Karsan 2020	Is there a place for Artificial Intelligence within patient triage?	General	A few words or sentences		Y							
**2025**	Keat 2025	PGxQA: A Resource for Evaluating LLM Performance for Pharmacogenomic QA Tasks.	LLM	A few words or sentences			Y				Y		
**2023**	Keller 2023	Comparing risk prediction models aimed at predicting hospitalizations for adverse drug events in community dwelling older adults: a protocol paper.	ML	A few words or sentences	Y								
**2025**	Kiyomiya 2025	Medication counseling for OTC drugs using customized ChatGPT-4: Comparison with ChatGPT-3.5 and ChatGPT-4o.	LLM	A few words or sentences			Y		Y				
**2023**	Kleebayoon 2023	Correspondence on Chat GPT-4, GPT-3.5 and drug information queries.	LLM	A few words or sentences	Y		Y						
**2024**	Kleebayoon 2024	Performance and risks of ChatGPT used in drug information: comment	LLM	The paper is focused on risk			Y		Y				
**2024**	Koch 2024	How Real-World Data Can Facilitate the Development of Precision Medicine Treatment in Psychiatry	General	A few words or sentences		Y							
**2024**	Koh 2024	The role of an artificial intelligence model in antiretroviral therapy counselling and advice for people living with HIV.	LLM	A few words or sentences			Y						
**2025**	Koh 2025	Continuing the Chat: How Can We Improve the Performance of an Artificial Intelligence Chatbot in Answering Clinical Infectious Diseases Pharmacotherapy Questions?.	LLM	A few words or sentences			Y						
**2021**	Koteluk 2021	How Do Machines Learn? Artificial Intelligence as a New Era in Medicine.	General	A few words or sentences	Y		Y						
**2023**	Kotsis 2023	Expectation of clinical decision support systems: a survey study among nephrologist end-users	ML	A few words or sentences	Y		Y						
**2024**	Kufel 2024	Let's Have a Chat: How Well Does an Artificial Intelligence Chatbot Answer Clinical Infectious Diseases Pharmacotherapy Questions?.	LLM	A dedicated section			Y						
**2025**	Kufel 2025	Reply: "Continuing the Chat: How Can We Improve the Performance of an Artificial Intelligence Chatbot in Answering Clinical Infectious Diseases Pharmacotherapy Questions?".	LLM	A few words or sentences			Y						
**2024**	Kuk 2024	Limitations of GPT-4 as a geriatrician in geri-oncology case conference: A case series	LLM	A few words or sentences			Y						Y
**2025**	Kumar 2025	Next-Gen Post-Diagnostic Care through Centralized AI and Smart Wearable Devices for Chronic Disease Management: A Narrative Review	Health monitoring system	A few words or sentences		Y	Y						

**2023**	Kunitsu 2023	The Potential of GPT-4 as a Support Tool for Pharmacists: Analytical Study Using the Japanese National Examination for Pharmacists	LLM	A few words or sentences	Y	Y	Y						
**2021**	Lam 2021	Machine Learning as a Precision-Medicine Approach to Prescribing COVID-19 Pharmacotherapy with Remdesivir or Corticosteroids.	ML	A few words or sentences			Y						
**2024**	Le 2024	Evaluation of health care decision-maker perspectives on artificial intelligence workflow implementation in health care and its utilization in the formulary decision-making process	General	A few words or sentences		Y							
**2023**	Lee 2023	Derivation and validation of a risk prediction score for nonsteroidal anti-inflammatory drug-related serious gastrointestinal complications in the elderly.	ML	A few words or sentences			Y						
**2021**	Lester 2021	Human versus machine editing of electronic prescription directions.	LLM	A few words or sentences			Y						
**2015**	Li 2015	An end-to-end hybrid algorithm for automated medication discrepancy detection	CDSS	A few words or sentences			Y						
**2024**	Li 2024	Engine of Innovation in Hospital Pharmacy: Applications and Reflections of ChatGPT.	LLM	A dedicated section		Y	Y						
**2024**	Li 2024	Integrated Knowledge Graph and Drug Molecular Graph Fusion via Adversarial Networks for Drug-Drug Interaction Prediction.	GNN	A few words or sentences				Y					
**2025**	Li 2025	Development and validation of a machine learning model to improve precision prediction for irrational prescriptions in orthopedic perioperative patients.	ML	A few words or sentences	Y								
**2003**	Lilienfeld 2003	Violation of homogeneity: a methodologic issue in the use of data mining tools.	General	A few words or sentences									
**2024**	Lima 2024	ChatGPT in Pharmacy Practice: Disruptive or Destructive Innovation? A Scoping Review	LLM	A dedicated section	Y	Y	Y						Y
**2023**	Liu 2023	Discrepancy between perceptions and acceptance of clinical decision support Systems: implementation of artificial intelligence for vancomycin dosing.	ML	A few words or sentences			Y	Y					
**2024**	Liu 2024	Large Language Models in the Clinic: A Comprehensive Benchmark	LLM	A few words or sentences		Y	Y						
**2024**	Liu 2024	Artificial Intelligence/Machine Learning: The New Frontier of Clinical Pharmacology and Precision Medicine.	General	A dedicated section	Y	Y	Y	Y					
**2024**	Loo 2024	Levodopa-induced dyskinesia in Parkinson's disease: Insights from cross-cohort prognostic analysis using machine learning.	ML	A few words or sentences	Y	Y	Y						
**2024**	Lorenz 2024	Quality of Drug Allergy Information Obtained Via An Artificial Intelligence Bot Versus Clinical Guidelines	LLM	The paper is focused on risk			Y						
**2024**	Lu 2024	Role of artificial intelligence in pharmaceutical health care	General	A few words or sentences	Y		Y						

**2024**	Luchen 2024	Pharmacy Futures: Summit on Artificial Intelligence in Pharmacy Practice.	General	The paper is focused on risk	Y	Y	Y			Y			
**2024**	Lucijanic 2024	The future is now, old man.	General	A few words or sentences			Y						
**2025**	Lv 2025	Machine learning-based prediction of vancomycin concentration after abdominal administration in patients with peritoneal dialysis-related peritonitis.	ML	A few words or sentences			Y						
**2024**	Ma 2024	Development and validation of an automatic machine learning model to predict abnormal increase of transaminase in valproic acid-treated epilepsy.	ML	A few words or sentences	Y								
**2025**	Mahdavi 2025	Multiomics and Artificial Intelligence for Personalized Nutritional Management of Diabetes in Patients Undergoing Peritoneal Dialysis	General	A few words or sentences	Y	Y	Y						
**2024**	Marques 2024	Advancing Precision Medicine: A Review of Innovative In Silico Approaches for Drug Development, Clinical Pharmacology and Personalized Healthcare.	General	A few words or sentences	Y	Y							
**2020**	Mathur 2020	Artificial Intelligence, Machine Learning, and Cardiovascular Disease	General	A dedicated section	Y	Y	Y						
**2024**	McMahon 2024	Automating untruths: ChatGPT, self-managed medication abortion, and the threat of misinformation in a post-Roe world	LLM	The paper is focused on risk			Y						
**2024**	Mediboina 2024	Assessing the Accuracy of Information on Medication Abortion: A Comparative Analysis of ChatGPT and Google Bard AI.	LLM	A dedicated section	Y	Y	Y						
**2020**	Meneveau 2020	Predicting adjuvant endocrine therapy initiation and adherence among older women with early-stage breast cancer.	ML	A few words or sentences			Y						
**2023**	Mesnier 2023	Relevance of medical information obtained from ChatGPT: Are large language models friends or foes?	LLM	The paper is focused on risk	Y		Y						
**2024**	Mogan 2024	Innovative and best models of palliative and end-of-life care - with focus on rural and remote communities.	General	A few words or sentences		Y							Y
**2024**	Mohammed 2024	Psychometric Properties and Assessment of Knowledge, Attitude, and Practice Towards ChatGPT in Pharmacy Practice and Education: a Study Protocol.	LLM	A few words or sentences	Y	Y							
**2023**	Montastruc 2023	Will artificial intelligence chatbots replace clinical pharmacologists? An exploratory study in clinical practice.	LLM	A few words or sentences	Y		Y						
**2002**	Moon 2002	A preliminary evaluation of neural network analysis for pharmacodynamic modeling of the dosing of the hydroxymethylglutaryl coenzyme A-reductase inhibitors simvastatin and atorvastatin.	CDSS	A few words or sentences			Y						
**2024**	Morath 2024	Performance and risks of ChatGPT used in drug information: an exploratory real-world analysis.	LLM	The paper is focused on risk			Y		Y				

**2024**	Mortlock 2024	Generative artificial intelligence (Gen-AI) in pharmacy education: Utilization and implications for academic integrity: A scoping review.	LLM	A few words or sentences			Y						
**2023**	Mosleh 2023	Medicine and Pharmacy Students' Knowledge, Attitudes, and Practice regarding Artificial Intelligence Programs: Jordan and West Bank of Palestine.	General	A few words or sentences			Y						
**2024**	Most 2024	Assessing the potential of ChatGPT-4 to accurately identify drug-drug interactions and provide clinical pharmacotherapy recommendations	LLM	A few words or sentences			Y						
**2024**	Mottaghi-Dastjerdi 2024	Advancements and Applications of Artificial Intelligence in Pharmaceutical Sciences: A Comprehensive Review.	General	A few words or sentences	Y	Y	Y	Y					
**2024**	Munir 2024	Evaluation of ChatGPT as a Tool for Answering Clinical Questions in Pharmacy Practice.	LLM	A dedicated section	Y	Y	Y						
**2024**	Murthi 2024	Evaluating EHR-Integrated Digital Technologies for Medication-Related Outcomes and Health Equity in Hospitalised Adults: A Scoping Review.	General	A few words or sentences									
**2024**	Murugan 2024	Empowering personalized pharmacogenomics with generative AI solutions.	LLM	A few words or sentences	Y		Y						
**2020**	Nelson 2020	Demystifying artificial intelligence in pharmacy.	General	A few words or sentences	Y		Y						
**2024**	Nelson 2024	Artificial intelligence and the future of pharmacy.	General	A few words or sentences			Y						
**2023**	Noviani 2023	A LITERATURE REVIEW: CHATGPT, WHAT WILL THE FUTURE OF PHARMACY PRACTICE BRING-A THREAT OR A BENEFIT?	General	A few words or sentences			Y		Y				
**2011**	Nunn 2011	Vancomycin Dosing Assessment of Time to Therapeutic Concentration and Predictive Accuracy of Pharmacokinetic Modeling Software	CDSS	A few words or sentences			Y						
**2019**	Nwosu 2019	Robotic technology for palliative and supportive care: Strengths, weaknesses, opportunities and threats	Robotics	A dedicated section	Y	Y							
**2025**	Okati 2025	Mobile applications on app stores for deprescribing: A scoping review	General	A few words or sentences			Y						
**2024**	Okpete 2024	Challenges and prospects in bridging precision medicine and artificial intelligence in genomic psychiatric treatment.	ML	A dedicated section	Y	Y							
**2024**	Omidian 2024	Synergizing blockchain and artificial intelligence to enhance healthcare.	General	A dedicated section	Y	Y		Y					
**2024**	Ong 2024	Generative AI and Large Language Models in Reducing Medication Related Harm and Adverse Drug Events - A Scoping Review	LLM	A few words or sentences	Y	Y							
**2024**	Oppenheimer 2024	Use of Technology to Improve Adherence in Allergy/Immunology.	General	A few words or sentences		Y	Y						

2024	Orok 2024	Pharmacy students' perception and knowledge of chat-based artificial intelligence tools at a Nigerian University.	General	A few words or sentences	Y	Y	Y						
2023	Oyebode 2023	Machine Learning Techniques in Adaptive and Personalized Systems for Health and Wellness	ML	A few words or sentences			Y						
2024	Ozturk 2024	Is ChatGPT reliable and accurate in answering pharmacotherapy-related inquiries in both Turkish and English?.	LLM	A few words or sentences			Y						
2024	Pais 2024	Large language models for preventing medication direction errors in online pharmacies	LLM	The paper is focused on risk			Y						
2024	Palaniappan 2024	Global Regulatory Frameworks for the Use of Artificial Intelligence (AI) in the Healthcare Services Sector.	General	A few words or sentences		Y							
2023	Pall 2023	Predicting drug shortages using pharmacy data and machine learning.	ML	A few words or sentences			Y						
2024	Pandya 2024	Causality Assessment of Adverse Drug Reaction Toxic Epidermal Necrolysis With the Aid of ChatGPT: A Case Report.	LLM	A few words or sentences			Y						
2024	Pandya 2024	Knowledge, Attitude and Practice of Artificial Intelligence Among Healthcare Professionals at a Tertiary Care Teaching Hospital in South Gujarat.	General	A few words or sentences	Y	Y						Y	Y
2020	Paranjape 2020	Short Keynote Paper: Mainstreaming Personalized Healthcare-Transforming Healthcare through New Era of Artificial Intelligence	General	A few words or sentences		Y	Y						
2023	Patel 2023	Perspectives of ChatGPT in Pharmacology Education, and Research in Health Care: A Narrative Review	LLM	A few words or sentences	Y		Y						
2021	Perkins 2021	Type 1 diabetes glycemic management: Insulin therapy, glucose monitoring, and automation.	ML	A few words or sentences			Y						
2024	Perrella 2024	Bridging the gap in AI integration: enhancing clinician education and establishing pharmaceutical-level regulation for ethical healthcare.	General	A few words or sentences	Y	Y							
2019	Peterson 2019	Machine Learning, Predictive Analytics, and Clinical Practice: Can the Past Inform the Present?	CDSS	A few words or sentences			Y						
2024	Petkovic 2024	Spotlight commentary: Integrating artificial intelligence in clinical pharmacology: Opportunities, challenges and ethical imperatives.	General	The paper is focused on risk	Y	Y	Y	Y					
2024	Pham 2024	ChatGPT's Performance in Cardiac Arrest and Bradycardia Simulations Using the American Heart Association's Advanced Cardiovascular Life Support Guidelines: Exploratory Study.	LLM	A dedicated section	Y		Y						
2024	Piffari 2024	AI Applications in the Healthcare Logistics and Supply Chain Sectors	General	A few words or sentences		Y							
2023	Poonprasartporn 2023	Comparing the accuracy and referencing of ChatGPT's responses to herbal medicine queries: A zero-shot versus roleplay prompting approach	LLM	A few words or sentences			Y						

**2024**	Popa 2024	Artificial Intelligence and medical specialties: support or substitution?	General	A few words or sentences	Y	Y							
**2023**	Poweleit 2023	Artificial Intelligence and Machine Learning Approaches to Facilitate Therapeutic Drug Management and Model-Informed Precision Dosing.	ML	A few words or sentences	Y								
**2022**	Prada-Ramallal 2022	Artificial intelligence in clinical pharmacology: Applications and limitations	General	A few words or sentences	Y								
**2024**	Putz 2024	Exploring the Capabilities and Limitations of Large Language Models for Radiation Oncology Decision Support	LLM	A dedicated section		Y	Y						
**2024**	R 2024	Enhancing Drug Discovery and Patient Care through Advanced Analytics with The Power of NLP and Machine Learning in Pharmaceutical Data Interpretation	LLM	A few words or sentences	Y	Y							
**2024**	R 2024	A comprehensive health assessment approach using ensemble deep learning model for remote patient monitoring with IoT.	ML	A few words or sentences		Y		Y					
**2023**	Raju 2023	Telemedicine - Application in Cardiology.	General	A few words or sentences		Y	Y						
**2024**	Ramasubramanian 2024	Comparative evaluation of artificial intelligence systems' accuracy in providing medical drug dosages: A methodological study.	LLM	A few words or sentences	Y	Y	Y						
**2024**	Rammal 2024	AI-Driven pharmacy practice: Unleashing the revolutionary potential in medication management, pharmacy workflow, and patient care	General	A dedicated section	Y								Y
**2023**	Rani 2023	Optimizing healthcare system by amalgamation of text processing and deep learning: a systematic review.	LLM	A few words or sentences		Y							
**2024**	Rao 2024	Proactive Polypharmacy Management Using Large Language Models: Opportunities to Enhance Geriatric Care.	LLM	A few words or sentences	Y		Y						
**2024**	Rao 2024	ChatGPT: A Conceptual Review of Applications and Utility in the Field of Medicine.	LLM	A dedicated section	Y	Y	Y		Y				Y
**2021**	Rodríguez 2021	Assisting older adults with medication reminders through an audio-based activity recognition system	ML	A few words or sentences		Y							
**2024**	Roosan 2024	Effectiveness of ChatGPT in clinical pharmacy and the role of artificial intelligence in medication therapy management.	LLM	A few words or sentences	Y	Y	Y						Y
**2024**	Ryan 2024	Artificial intelligence and machine learning for clinical pharmacology.	General	A few words or sentences	Y		Y						
**2023**	Sallam 2023	ChatGPT applications in medical, dental, pharmacy, and public health education: A descriptive study highlighting the advantages and limitations.	LLM	A few words or sentences	Y	Y	Y						

**2025**	Samajdar 2025	Artificial Intelligence in Healthcare: Current Trends and Future Directions.	General	A few words or sentences	Y	Y							
**2025**	Sangam 2025	The role of digital health in the management of warfarin therapy	General	A few words or sentences	Y	Y							
**2024**	Sarris 2024	Artificial intelligence and psychedelic medicine	General	A dedicated section	Y	Y	Y						
**2020**	Senbekov 2020	The recent progress and applications of digital technologies in healthcare: A review	General	A few words or sentences		Y				Y			
**2022**	Sharma 2022	Development and Validation of a Machine Learning Model to Estimate Risk of Adverse Outcomes Within 30 Days of Opioid Dispensation.	ML	A few words or sentences			Y						
**2024**	Shin 2024	Performance of the ChatGPT large language model for decision support in community pharmacy.	LLM	A few words or sentences		Y	Y						
**2021**	Sirois 2021	Exploring polypharmacy with artificial intelligence: data analysis protocol.	LLM	A few words or sentences		Y		Y					
**2024**	Smoke 2024	Artificial intelligence in pharmacy: A guide for clinicians.	General	A dedicated section	Y	Y	Y						
**2025**	Smoke 2025	The two dimensions of pharmacy artificial intelligence tools	General	A few words or sentences			Y						
**2023**	Snoswell 2023	Pharmacist vs machine: Pharmacy services in the age of large language models	LLM	The paper is focused on risk	Y	Y	Y						
**2024**	Sridharan 2024	Unlocking the potential of advanced large language models in medication review and reconciliation: A proof-of-concept investigation	LLM	A few words or sentences			Y						
**2024**	Sridharan 2024	Investigating the capabilities of advanced large language models in generating patient instructions and patient educational material.	LLM	A few words or sentences			Y						
**2024**	Stanceski 2024	The quality and safety of using generative AI to produce patient-centred discharge instructions	LLM	A few words or sentences			Y						
**2024**	Stevens 2024	Lightening the Load: Generative AI to Mitigate the Burden of the New Era of Obesity Medical Therapy.	LLM	A few words or sentences	Y	Y	Y						
**2025**	Stroop 2025	Assessing GPT-4's accuracy in answering clinical pharmacological questions on pain therapy.	LLM	A dedicated section		Y	Y	Y					
**2024**	Suffoletto 2024	Deceptively Simple yet Profoundly Impactful:Text MessagingInterventions to Support Health	LLM	A few words or sentences		Y	Y						
**2024**	Sumner 2024	Developing an Artificial Intelligence-Driven Nudge Intervention to Improve Medication Adherence: A Human-Centred Design Approach	General	A few words or sentences		Y							
**2023**	Sun 2023	AI in healthcare: navigating opportunities and challenges in digital communication.	General	A dedicated section	Y	Y		Y					
**2024**	Tade 2024	Artificial Intelligence in the Paradigm Shift of Pharmaceutical Sciences: A Review	General	A few words or sentences		Y							

**2024**	Taha 2024	Perception, concerns, and practice of ChatGPT among Egyptian pharmacists: a cross-sectional study in Egypt.	LLM	A few words or sentences	Y	Y	Y	Y					
**2025**	Tangsrivimol 2025	Benefits, limits, and risks of ChatGPT in medicine	LLM	A dedicated section	Y	Y	Y						Y
**2024**	Thacharodi 2024	Revolutionizing healthcare and medicine: The impact of modern technologies for a healthier future-A comprehensive review	General	A dedicated section	Y	Y	Y						
**2024**	Tilva 2024	A REVIEW ON SCOPE OF ARTIFICIAL INTELLIGENCE IN PHARMA	General	A few words or sentences	Y	Y	Y						
**2024**	Triplett 2024	A comparison of drug information question responses by a drug information center and by ChatGPT.	LLM	A few words or sentences	Y		Y						
**2023**	Ullah 2023	Smart Technologies Used as Smart Tools in the Management of Cardiovascular Disease and Their Future Perspective	General	A few words or sentences	Y	Y	Y			Y			
**2024**	vanDellen 2024	Precision psychiatry: predicting predictability.	ML	A dedicated section	Y		Y						
**2024**	vanNuland 2024	Assessing accuracy of ChatGPT in response to questions from day to day pharmaceutical care in hospitals.	LLM	A dedicated section			Y		Y				
**2018**	Vayena 2018	Digital Medicine and Ethics: Rooting for Evidence.	General	A few words or sentences	Y	Y							
**2024**	Venerito 2024	Large language models: rheumatologists' newest colleagues?	LLM	A few words or sentences	Y		Y						
**2024**	Venkatesh 2024	Health Digital Twins in Life Science and Health Care Innovation.	General	A few words or sentences	Y	Y							
**2024**	Vordenberg 2024	Investigating Older Adults' Perceptions of AI Tools for Medication Decisions: Vignette-Based Experimental Survey.	LLM	A few words or sentences	Y								
**2025**	Warraich 2025	FDA Perspective on the Regulation of Artificial Intelligence in Health Care and Biomedicine	General	A few words or sentences	Y	Y	Y						
**2020**	Wehbe 2020	Predicting High-Risk Patients and High-Risk Outcomes in Heart Failure	ML	A dedicated section	Y		Y						
**2024**	Westbrook 2024	Using AI to improve medication safety	General	A dedicated section	Y		Y						
**2023**	Wong 2023	Role of artificial intelligence in pharmacy practice: A narrative review	General	A few words or sentences	Y								
**2025**	Wong 2025	Forecasting the impact of artificial intelligence on clinical pharmacy practice	General	A dedicated section		Y	Y						
**2024**	Xu 2024	Large language models management of complex medication regimens: a case-based evaluation	LLM	A dedicated section			Y						
**2022**	Yoon 2022	Machine learning in medicine: should the pursuit of enhanced interpretability be abandoned?.	General	The paper is focused on risk	Y			Y	Y				
**2025**	Young 2025	Racial, ethnic, and sex bias in large language model opioid recommendations for pain management	LLM	The paper is focused on risk	Y								
**2024**	Yu 2024	Flexit: Flexible Location Encoding for Item Access in Medication Management Systems	Robotics	A dedicated section		Y	Y						

**2023**	Zawiah 2023	ChatGPT and Clinical Training: Perception, Concerns, and Practice of Pharm-D Students.	LLM	A few words or sentences	Y	Y	Y						
**2024**	Zhang 2024	Student Pharmacists' Perceptions of Artificial Intelligence and Machine Learning in Pharmacy Practice and Pharmacy Education.	General	A few words or sentences	Y		Y						
**2024**	Zhu 2024	Advancing Glaucoma Care: Integrating Artificial Intelligence in Diagnosis, Management, and Progression Detection.	General	A dedicated section	Y		Y	Y					
**2023**		Executive summary of the meeting of the 2023 ASHP Commission on Goals: Optimizing Medication Therapy Through Advanced Analytics and Data-Driven HealthcareA1 - Anonymous.	General	A few words or sentences	Y		Y						

Table S3: Data Charting for Grey Literature Reports					**Risks described**								
**Year**	**Pharmacy organization**	**Title**	**AI tool/system investigated**	**Extent of risks reported**	**Bias**	**Privacy/ Security**	**Inaccuracies**	**Transparency (Black Box)**	**Reproducibility**	**Accessibility**	**Sycophancy**	**Outdated**	**Patient-Provider Relationship**
2026	APhA	AHA Response to HHS RFI on AI in Health Care	General	A few words or sentences	Y	Y	Y						
2026	APhA	RE: [RIN 0955-AA09] Health Data, Technology, and Interoperability: ASTP/ONC Deregulatory Actions To Unleash Prosperity	General	A few words or sentences		Y	Y	Y					
2026	EAHP	USING ARTIFICIAL INTELLIGENCE TO TRAIN FUTURE PHARMACISTS : EVALUATION OF A CHATBOT DEVELOPED FOR THE ONCOLOGY PHARMACY UNIT	LLM	A few words or sentences			Y					Y	
2026	FIP	PSWE-09 - Navigating ethical and regulatory considerations of artificial intelligence (AI) in pharmacy practice	General	A few words or sentences	Y	Y							
2025	FIP	FIP STATEMENT OF POLICY Artificial intelligence in pharmacy practice	General	A few words or sentences	Y		Y		Y				
2025	FIP	An artificial intelligence toolkit for pharmacy. An introduction and resource guide for pharmacists	General	A dedicated section	Y	Y		Y	Y				
2024	ASHP	Ambulatory Care Practitioners Artificial Intelligence: A Primer	General	A dedicated section	Y	Y			Y				
